# A qualitative analysis of factors impacting resilience among youth in post-conflict Liberia

**DOI:** 10.1186/s13034-016-0114-7

**Published:** 2016-08-12

**Authors:** Elizabeth J. Levey, Claire E. Oppenheim, Brittany C. L. Lange, Naomi S. Plasky, Benjamin L. Harris, G. Gondah Lekpeh, Isaac Kekulah, David C. Henderson, Christina P. C. Borba

**Affiliations:** 1 The Chester M. Pierce, MD Division of Global Psychiatry, Massachusetts General Hospital, 5 Longfellow Place, Boston, MA 02114 USA; 2 Harvard Medical School, 25 Shattuck St., Boston, MA 02115 USA; 3 University of Illinois College of Medicine, 1853 West Polk St, Chicago, IL 60612 USA; 4 Department of Psychiatry, Boston Medical Center, 840 Harrison Ave, Boston, MA 02118 USA; 5 Department of Social Policy and Intervention, University of Oxford, Barnett House, 32-37 Wellington Square, Oxford, UK; 6 Johns Hopkins Bloomberg School of Public Health, 615 North Wolfe St, Baltimore, MD 21205 USA; 7 A.M. Dogliotti College of Medicine, University of Liberia, Monrovia, Liberia; 8 Boston University School of Medicine, 72 East Concord St, Boston, MA 02118 USA

**Keywords:** Liberia, Post-conflict, Qualitative analysis, Youth, Adolescent, Resilience

## Abstract

**Background:**

In 2008, 5 years after the Liberian civil war ended, there were an estimated 340,000 orphans in Liberia, 18 % of the total child population of the country. Given that children make up half the population and that these children experienced significant trauma and loss both through direct exposure to the war and then to the Ebola epidemic, and indirectly as a result of the trauma experienced by their parents, the recovery of these children is essential to the recovery of the nation as a whole. The goal of this research was to identify factors contributing to resilience among youth in post-conflict Liberia. Resilience was defined as evidence of adaptive functioning and psychological health.

**Methods:**

Seventy-five young people (age 13–18) in the capital city of Monrovia, Liberia were recruited in 2012. Semi-structured interviews were conducted, and demographic data were collected. Interviews were then transcribed and coded thematically.

**Results:**

Forty-six of the participants were attending school, and 29 were not enrolled in school. Youth enrolled in school demonstrated greater adaptive functioning. This was particularly true for boys in any school setting and girls attending private school. Youth not attending school were more likely to have lost family members or become estranged from them, and many were also engaging in substance use. Emotion regulation, cognitive flexibility, agency, social intelligence and, in some cases, meaning-making were found in participants who showed resilient outcomes.

**Conclusions:**

Caregiver relationships mediate the development of psychological capacities that impact resilience. These findings suggest that youth who have lost a caregiver, many of whom are not attending school, are experiencing a significant ongoing burden in terms of their daily functioning and psychological health in the post-war period and should be the focus of further study and intervention targeting substance use and community reintegration.

*Trial registration* Partners Healthcare IRB Protocol# 2012P000367.

## Background

Post-conflict societies invariably struggle to re-establish order while at the same time facing major health consequences of the trauma experienced by their populations. In addition to medical issues, these consequences frequently include mental illnesses such as post-traumatic stress (PTSD), mood, and substance use disorders. A meta-analysis of 17 studies of children in post-conflict societies from around the world found that PTSD, depression, and anxiety were all increased in this population [1].

In the post-conflict setting, there has been an increasing focus on resilience, which is defined in different ways. Functioning, including income generation and educational attainment, contributes to resilient outcomes, but it is only part of the picture. Psychological health, in terms of subjective sense of wellbeing and safety and absence of unmanageable stress, is also an important factor [2–5]. Sense of coherence, the extent to which people are confident that their environment is predictable and that things will work out as well as can reasonably be expected, has been used in some studies as a measure of psychological recovery post-conflict [6, 7]. This is particularly relevant following the disorganizing impact of war.

A number of studies have sought to identify factors common to resilient individuals. Based on their work with non-traumatized clinical populations in the United States, Southwick and his collaborators have found resilient individuals to be capable of emotion regulation and cognitive flexibility [8]. They have a sense of agency and optimism, and they engage in active coping and identify themselves as spiritual [8]. Social intelligence and the ability to connect are also linked to resilience. Sociability was associated with resilience in a population of teenage mothers in Kauai. Those who were able to form connections outside their families were doing better financially and in terms of relationship stability as adults [5].

A few studies have examined protective factors specifically in children in post-conflict settings. One study examined the sense of coherence among children and adolescents who lived through the war in Croatia. Childhood stability, acceptance of one’s own ethnic identity, management of uncertainty, and finding meaning in work emerged as strengths that contribute to a sense of coherence [7]. A study of former child soldiers in Colombia found that the individual factors most consistently associated with resilience were agency, empathy, affect regulation, hope, spirituality, and morality [4]. A study of Rwandan key informants identified the following protective factors among children of families affected by genocide and HIV: perseverance, self esteem, family unity, good parenting, and community cohesion [3].

None of the studies referenced above included data from Liberia. Between 1989 and 2003, the country experienced a brutal civil war characterized by ethnic killings, sexual violence and the use of child soldiers. The war left more than 800,000 people displaced, of a total population of 3.5 million, and it destroyed the productive capacity and physical infrastructure of the country, and eroded family and community ties [9, 10]. Liberia has a young population; 5 years after the war ended, 50 % of the population was under the age of 18 [10]. There were an estimated 340,000 orphans in Liberia, 18 % of the total child population of the country [10]. Given that children make up half the population and that these children have experienced significant trauma and loss both through direct exposure to the war and then to the Ebola epidemic and indirectly as a result of the trauma experienced by their parent, the recovery of these children is essential to the recovery of the nation as a whole.

In the wake of this trauma, access to mental health care is limited. There is only one psychiatrist living and practicing in the country. There is no psychiatric residency training program and no training available for clinical psychologists. A survey of households and healthcare facilities in rural Nimba County, Liberia found that while all of the hospitals and most health clinics provided malaria and HIV care, none of the hospitals and only 11 % of the clinics offered mental health care [11].

The Liberia needs assessment survey sought to characterize and quantify the mental health burden of the war on the children of Liberia [12]. Key informants were asked to describe the most emotionally disturbing events or experiences to have affected young Liberians in the past 20 years and how best to help specific vulnerable groups. Youth were perceived to be experiencing significant adverse emotional, behavioral and functional outcomes related to exposure to war and its aftermath. The key informants suggested that needs included professional mental health services for some children, as well as greater community support [12, 13].

This study was undertaken in order to capture the needs of Liberian adolescents in their own words and identify the way forward. The goal of this research is to identify factors contributing to resilience among children in post-conflict Liberia. We will assess for the presence of these factors in the participants we interviewed and examine how they interact to promote resilience and adaptive functioning. Considering the findings in the literature reviewed and the observations made about resilience in children exposed to violence, we will define resilience as evidence of daily functioning, ability to make realistic plans for the future, and presentation of an overall consistent and coherent narrative.

## Methods

### Study procedures and participants

A qualitative research design was used with a purposive sample of 75 participants. In-depth interviewing was chosen as the data collection method in order to best capture the personal narratives of individual children [14]. The interview guide was designed and implemented by the researchers using both a deductive and an inductive conceptual framework. The initial interview guide was developed deductively based upon the existing literature, with input from Liberian collaborators. Prompts were developed to assess factors that had already been identified to be associated with resilience. Further revisions were made after each interview to refine and deepen the questions. Oversight, guidance and approval was provided by the University of Liberia Institutional Review Board (IRB) and the Partners Healthcare IRB.

Participants were recruited from February 13th through March 1st and December 4th through December 21st, 2012. The initial group was recruited from three schools and the subsequent group of youth not attending school was recruited from neighborhoods in Monrovia and the surrounding area. All children ages 13–18 were eligible to participate, and an effort was made to recruit both males and females. The Republic of Liberia’s Ministry of Education identified three large, well-attended schools in three distinct areas within Monrovia. The participating schools included two government schools (a junior high school and a senior high school) and a private senior high school. These schools were chosen in order to capture distinct age and socioeconomic groups. At each school, the principal notified the students of the opportunity to participate in the research study and answer questions about school, family, and past experiences. Children under 18 had to obtain consent from a parent or guardian and then give their assent to participate. Those who were 18 consented for themselves. Those students who returned signed consent forms first were interviewed.

The research team then recruited and interviewed children not attending school. They were identified in two different communities in Monrovia, with the help of community leaders. Both communities had significant problems with poverty, crime, and substance use, and many of the children were not attending school. The leaders went to a few areas in their communities where youth commonly gathered and explained that there was an opportunity to participate in a research study. They distributed printed information and consent forms and told the young people when and where the interviews would take place. They helped to organize a loose schedule with approximate days and times for each child to come. The leaders were present at the building while the interviews were conducted but were not present in the room during the interviews.

Initially, a $5 USD phone card was agreed upon by the research team and the IRB as an appropriate compensation for participation. After the initial phase of recruitment, the research team observed that this was a significant inducement to participate. At the suggestion of the IRB, the research team changed the compensation to $2 USD. Any child identified as having acute psychiatric needs was referred to the study physician, a Liberian child psychiatrist.

### Data collection

Primary data was collected using semi-structured interviews averaging 1 h in length. Interviews were conducted by the principal investigator together with a Liberian medical student, who acted as both a linguistic and cultural interpreter. The interviews were conducted in a private area. Student participants were interviewed on school grounds. Interviews were conducted for three to 4 days at each school. Those not attending school were interviewed in an office where the community leader worked. Interviews were digitally recorded, and notes were taken by the interviewers regarding participants’ affect and non-verbal communication during the interview. At the end of each day, recordings and notes were reviewed and revisions were then made to the interview guide. The interviews were transcribed by Liberians living in the United States.

### Analysis

A directed content analysis was used because there was an existing body of literature about factors impacting resilience in youth, but there was insufficient data about the specific population that was studied [15]. Coding was performed by four coders who worked separately and sequentially over the course of 2 years, beginning 6 months after data collection was initiated. While coding the first five interviews, the coders met after each interview to compare their findings. There was a discussion about topics that were raised by multiple participants and whether they were saying the same thing or articulating distinct themes. Codes developed and used by multiple coders were then added to the codebook. When there was a discrepancy, coders explored it in an effort to uncover the deeper meaning at the heart of the theme. Discussion continued until a consensus was reached. Coders then met after every five interviews to further refine the codebook. Once there was a consensus as to the codes and definitions being used, all interviews were recoded. Then the themes and definitions were further refined.

Both inductive and deductive approaches were used [14]. First, manifest content was grouped thematically. Thematic groupings were then labeled, and these group labels were used to generate broad themes. These broad, overarching themes were divided into sub-themes. Within each theme and sub-theme, the researchers drew comparisons, looking for overlap and differences, as well as newly emerging topics and patterns. Themes identified included agency, altruism, responsibility, corruption, uncertainty, family and community relationships, and exposure to violence. Responses were reviewed to identify each theme and sub-theme. The analysis was conducted using grounded theory, memoing, and coding [14]. NVivo was employed for data management [16].

## Results

### Descriptive data

Seventy-five participants were interviewed. They reported being between 13 and 18 years of age. The average age of the participants was 16.4 years (standard deviation 1.8 years). There were 38 females and 37 males. There were 35 public school students, 11 private school students, and 29 children not attending school at the time of the interviews (see Table 1).

**Table 1:** Demographic characteristics of study participants (*n* = 75)

Characteristic	M (SD) or N (%)
Average age	16.4 (1.8)
Females	38 (51 %)
Males	37 (49 %)
Private school	11 (14.7 %)
Government school	35 (46.7 %)
Not attending school	29 (38.7 %)

### Analyzed data

In this population, resilience hinged upon children being able to regain a sense of stability in their lives. Essential to this were emotion regulation, cognitive flexibility, agency and social intelligence. Meaning-making also played a role in some cases. Children who could delay gratification, modulate affectively-charged experiences and self-soothe were effective at regulating their emotions without resorting to unhealthy coping strategies. Cognitive flexibility was seen in children who were able to put their experiences into perspective and thinking creatively about alternative strategies for managing difficult situations. An internal sense of control or agency was crucial and reinforced children’s self-esteem and optimism. Social intelligence was the individual factor that allowed children to access other sources of support in their changing circumstances. It included empathy, altruism, and the ability to form connections. Ability to connect was particularly important for children who had lost one or both parents, as it allowed them to form other supportive relationships in their communities.

### Emotion regulation

After the loss of family members and exposure to violence that many of these children faced, emotion regulation was essential for continued functioning. Children needed to be able to tolerate intense affective states and utilize healthy coping strategies when they were in distress. Those who could do so built confidence in their own self-regulatory capacity and were also able to maintain relationships that would have been jeopardized if they were to act out strong negative affects. These relationships were a source of support that was further stabilizing. Children who were able to regulate their emotions were generally living with their families and attending school. Those who were not with their families had formed other important relationships. They were involved in recreational activities. They felt supported in their relationships with family and peers:

“*Some of the things I saw, at times, it come to my memory, but I try to control myself. Because some of those people that were in the war, you find out majority of them crippled, some of them crazy… When I have such thinking coming around me at times, I go around friends, play with them so as I can erase that from my mind, because the more I think on it, the more I feel bad*.”

There was a notable difference between children who had a stable environment postwar and those who did not. Stable support figures in the present helped them to cope with difficult memories of the past. Children with emotionally dysregulated caregivers experienced episodes of dysregulation themselves:

*“Sometimes when I study I always forget because my mind is not really to the lesson, and as such everything don’t really register in my mind, so that caused me to fail in some test… Sometimes the teachers give you notes to go home and study and then your parents do something to you that you always think about so your mind will not be focus again so that will always cause me to fail… My mother is very harsh with me so when I do something to her at home, I am very afraid of my mom to come to her and say, ‘Mammie, I am sorry’… Even if I go to her, she won’t listen to me*.”

Then there were those who had lost their relationships with their caregivers, either due to death or separation, and were unable to form new supportive connections. They were overwhelmed by their emotions and resorted to maladaptive coping strategies, like substance use. Their relationships were based on engaging in substance use, rather than supporting their functioning:

*“When I think about my sister because she used to send me to a very good school. I was not involve with foolish friends, and after school I will go for study class and come home and cook, so after she died it played on me. First I involved into drinking and from there when I drink and I sleep I don’t think about her again. After that I started to smoke the grass [cannabis]. When I smoke the grass it makes me to eat and sleep, and then I don’t think too much.”*

While some children faced greater loss than others, it was not the losses themselves but whether they formed new relationships that made the difference in their ability to self-regulate. The relationships helped them to self-regulate, and those who had lost their primary caregivers and were able to self-regulate were more appealing to other adults.

### Cognitive flexibility

Cognitive flexibility was also associated with resilience. Children who could be self-reflective, use metaphor, and think creatively about their problems were able to identify more adaptive solutions and also maintain a sense of hope. These children varied in terms of age, gender, school attendance and parental status. They had all experienced serious losses and found ways of understanding themselves and their circumstances.

Self-reflection and perspective-taking was demonstrated by children who could describe the kind of people they were and how they were perceived by others. They gave nuanced descriptions, demonstrating that these ideas were carefully considered. One girl described her process for making decisions:

“*I have always been the person that will analyze things. When I have a problem, I will look at the good and bad side. I make my decision and then I go out there and ask for other people’s opinion. And I will ask two or three persons and what they will say to me and then I weigh it and then I make my own decisions*.”

Children like this recognized themselves as individuals distinct from other children. They could see that they were choosing to do things in a particular way, and if it was not working, they could make different choices.

Faced with brutal realities, children who were able to think creatively could imagine other possibilities, which gave them the hope to persevere. They also had a sense of balance between work and play, suffering and joy, which allowed them to withstand difficult experiences. One girl explained a geographic metaphor for the different pediatric wards at the hospital. The intensive care unit was “*Iraq*,” where most of the patients died. The regular inpatient unit was “*China*,” where the patients were seriously ill, but they had a chance. The outpatient clinic was “*America*,” where the patients were healthy. She explained that she would like to become a pediatrician and work in China:

“*I will like to take care of them in China because in China is much better. There is a chance that some may die but it will not be like in Iraq. You have the chance to know whether they will live or die, also unlike Iraq…. I want to be a doctor [because] most of our people are dying, and you find out people are not going in that field… I want to know it to take care of people*.”

Cognitive flexibility allowed children to make a realistic appraisal of the difficulties they faced and respond effectively. They were neither overly optimistic that things would work out, nor were they despairing. They saw their situations for what they were and tried their best to meet challenges with their strengths.

### Agency

Children demonstrated agency, a sense that they had control over their lives and the ability to impact their environment, by challenging themselves, showing determination in the face of obstacles and asserting their opinions and preferences. Agency was also mutually reinforcing with two other factors that contributed to resilience: self esteem and hope. A belief in their own self-efficacy supported children’s beliefs that they were of value and that they had reason to be hopeful about the future.

Some children attending school had many responsibilities, including schoolwork, household chores, and earning money. They worked hard in all of these areas because they believed they had the power to positively impact their futures. An 18-year-old girl commented:

“*Everyone makes mistakes, but some people if they make mistakes, they can just leave it just like that. They don’t love to fix it back, but for me, when I make a mistake, I love to at least go to someone to correct my mistake… It can make you learn, but if you make a mistake and you just sit by yourself, then you won’t get it*.”

Another way in which children demonstrated that they felt a sense of agency was by expressing opinions and preferences. They believed that their preferences mattered and that their opinions were heard. This was seen in boys attending school and girls attending private school. Their preferences concerned how they should be treated by authority figures at home and in school. They thought their parents should speak to them when they did something wrong, rather than beating them because beating “*will not teach you to correct your mistake; it will just make you afraid*.” In the school setting, they wanted teachers who would come to class, be engaged, and offer explanations. They had complaints about teachers requesting bribes in exchange for passing grades. One girl explained:

“*We try our best to study hard to make our grades, but when we make our grades, they try hard to take money from us. It’s not that the principal is in favor of it, but he doesn’t have control over it*.”

Agency also fostered a sense of self-esteem. Self-esteem was evident when children spoke about their abilities in academics and athletics and being seen as leaders by peers and adults. The children who described themselves as intelligent were boys attending school. They reported that they were passing all of their classes, even those that most students were failing. They had hope for the future and wanted to obtain advanced degrees and become teachers or other types of professionals. This example illustrates how a belief in one’s intelligence fosters self-esteem, which supports a hopeful, optimistic outlook and a sense of self-efficacy. An 18-year-old boy explained the following:

“*[Our teacher] knows the subject, and when he brings a test, it can’t be easy. If he brings the test he can say ‘if you pass in this test you are not a small person’… But sometimes when that man bring test, the question can be very tricky if you don’t use your third eye, which is the eye of your mind to solve that problem. I can make a successful pass, and my name has left there in history*.”

In contrast to these children who had a sense of their ability to impact the trajectory of their lives, there were others who felt stuck. They were not going to school or working. They felt that they were doing nothing and that there was nothing they could do to change their circumstances. A boy not attending school said:

“*I am not doing anything, so that can make me to feel bad. No family, no mother and no father so I can feel bad. I am still feeling bad because I have no opportunity, no helping hand, nobody to comfort me and nobody to shelter me*.”

Among this group of children not attending school, some expressed a vague hopefulness that they would be able to change things for themselves but without any idea of how this would be possible. Those who were using drugs believed they needed medical treatment in order to stop. A few children had stopped successfully and shifted their focus to going to school, but they did not know how they would be able to do this.

### Social intelligence

Social intelligence encompassed not only empathy and altruism but also an ability to develop relationships. Empathy and altruism were more common among children attending school. For those children not attending school, the ones who expressed empathy had more exposure to school or had educated caregivers who wanted to send them to school. Empathy was seen in both genders. Interest in helping professions was seen more commonly among girls and societal concern was seen more in boys.

Participants expressed empathy for family members, recognizing that while the participants themselves were struggling, those around them were also facing difficulties:

“*My sister doesn’t have anybody to support her. She’s supporting herself and at the same time supporting me… It is very difficult for her. It is also difficult for me because I am not supposed to come from school and go straight to the market to sell. And if I don’t sell, how will I go to school? So I have to*.”

A number of children who were attending school, as well as a few who were not, expressed interest in pursuing a career that would help others. Some children were interested in studying law or medicine: “*In the future I want to be a lawyer, to help people get their property back*.” While the school children had specific plans for careers they wished to pursue and reasons for choosing them, children not in school were motivated by the fantasy of being powerful and able to provide for others, rather than needing help themselves.

Most children spoke about their own personal concerns, but some also demonstrated altruism through their concerns about Liberia and its people. This was expressed in particular by boys attending school. They had their basic needs met, which allowed them to consider the wellbeing of others. They were concerned about poverty, access to education, and fostering development:

“*We are kindly appealing to the government to assist us. We are the students of this country. We are facing some difficulties. One is transportation. The government needs to provide some buses for we the students because in the morning when you get up, there’s no way to find a car and get on your campus, and now that’s why we trying to appeal to the government to help us with some buses*.”

Among the four children who had lost one parent and had no caregiver, two were able to form stabilizing connections in their communities. They both described supportive, loving relationships with the parent they lost before that parent died. The other parent was seen as ineffectual and unable to help. This relationship was distant but did not seem to be a source of anger or resentment. One of the participants was working to support herself and her children, and a neighbor offered to help her:

“*She can just help me. I can give her something when I have it, and when she cooks, she will take out food for them. She is selling right in front of the house so she can go nowhere. She told me to start selling, and she said ‘I will be helping you to look after your children’ so I don’t pay her*.”

Another participant was also forming connections for himself. His mother was killed during the war, and his father was paralyzed and living in a remote area. Before the war, he had been an exceptional student and skipped two grades. After the war, he was sleeping on the ground in market stalls. He was able to earn some money doing odd jobs at the market and saved enough to start a selling business. He could then afford to rent a room for himself and eventually returned to school. He had no family in Monrovia but found a woman in the community who helped him:

*“I can call my aunty, and I called her aunty because when I have problems, I can go to her… One time she was getting in a car, and I helped her to put her load in the car. She showed me her house because I explained some problems to her. So she told me, when I need help, I should go to her house… She can help me with food and sometimes money. She herself she doesn’t have it, but she can still help me.”*

This boy was the only study participant who was putting himself through school without any family support. It was by reaching out and offering to help someone else that he had found help for himself. While this woman had little to offer him in terms of financial resources, she gave him a sense of connection and support.

Those who did not form connections were struggling in their daily lives. They were using drugs and engaging in prostitution. They had relied heavily on the parent who died and did not have a good relationship with the living parent. They did not explicitly say why, but they indicated that their needs were not being met at home, so they chose to leave home and sought care from unreliable and unsafe people:

*“[My father] didn’t have time for [me] so everyone was doing their own thing at that time. So that’s how I ran away because I was not feeling good. He wanted to send me to school and ran away because I don’t have food to eat, and he wants me to go to school. So I said no and I ran away. It was better for me to be on my own, and I survived… I wanted to have my own money and do stuff for myself but he said no.”*

For this girl, her closest relationship was with her boyfriend. They had known each other for about a month, and their relationship was based on money and sex. She considered it different from prostitution because they had sex regularly and shared money more freely with each other. The only ambition she had for her future was to stop using drugs, but she had no plan for how to do so.

Another girl appeared more functional on the surface, but she was also struggling. She dropped out of school months before graduating. Although she was clearly interested in forming connections, she was not able to form and maintain connections with people in her environment in a way that she could use to better herself and reconnect with her family and community. Instead, she sought connections motivated by a kind of rescue fantasy that someone from the outside would be able to transform her life completely. She was interested in learning personal details about the interviewer, in an attempt to develop an ongoing relationship. She also described a female missionary who had come from the United States a few years prior. She still hoped that this woman would return and help her:

“*One American girl came, and I was with her, but after she left and went to the States. She was here for about two to three years, and she was teaching and a reverend. She interviewed me, and she took me and carried me. Her interview was only for men, but I was very lucky, and I was the only girl amongst all the girls, and I was living there with her… They took the address book with the woman address and everything in it. So I cannot even call her and I just sitting down now.*”

### Meaning

Children described a variety of systems of meaning: religious beliefs, traditional beliefs, and moral codes of behavior were among them. All of the children interviewed in this study endorsed having a religious identity, either Christian or Muslim. They prayed to God for good things to happen to them in the future. Many said things like, “*God will make everything possible. I know God will lift me up for my tomorrow*.” Upon closer examination, however, the children were actually saying quite different things. For some, their belief system contained the idea that their lives mattered, God loved them, and they had the power to make things happen for themselves. For others, their belief in a higher power reinforced their sense of helplessness. They could do nothing for themselves and instead had to wait for a more powerful being to rescue them from their suffering.

Children whose belief systems reinforced their agency showed more adaptive functioning. Only one participant, an 18-year-old boy, described finding meaning in suffering. He lost both of his parents and was not in school. He had been using drugs and spent time in jail for armed robbery. He stopped using drugs in jail and stayed clean after he was released. He then got a job and developed a relationship with a neighbor who paid him to do chores for her:

“*In jail I started to face many problems with my eyes. If I keep smoking, it will embarrass my eyes, and it will affect it more… God made it so I can go to jail so that I can stop smoking*.”

Children also spoke about traditional beliefs, including the concept of “African science,” a curse placed out of jealousy that brought serious harm or death. When someone died in a way that was not well understood, it was frequently attributed to African science:

“*My father was a very good man who loved to help people. According to the native doctor they went to, he said because he loved to help that’s why they sent African science on him…. Some people they will go to the native doctor for country medicine. They tell you how you should give it to the person for the person to die. Some people can put it in your drink, they can put it in your food, in water, anything they feel that you will be able to eat they can place it in*.”

African science was used to explain deaths by natural causes or sudden, unexplained illnesses, but it was not used to explain deaths due to war, which, at least superficially, were easier to understand. Children who were functioning well talked about African science, as did children who were struggling. Children were told by adults that a death was due to African science, but they did not understand it; it merely added another layering of mystery over a mysterious event.

Finally, meaning was described in terms of moral codes of behavior. For some children, obedience and deference to authority were valued:

“*He can punish us by beating us. I don’t feel bad because I know he wants the best out of us that’s why he punishing us. Even though I can cry, but then I can realize that he taking us from the wrong way and bring us the right way*.”

Others valued their autonomy and being treated respectfully by adults. They explained that advising a child (speaking to him about what he did wrong) was better for the child and more effective than beating him because then the child would develop an internal sense of right and wrong:

“*You cannot teach a child by beating. Too much beating is not good. Is good at times you set the child down and advise them. Every human being has what they call conscience, at least sometime your conscience will beat you and you forget about what you been doing and at least go on the rightful path*.”

For these children, their ideas about meaning supported their sense of agency and their ability to impact their own lives and the lives of others.

## Discussion

**Fig. 1:**
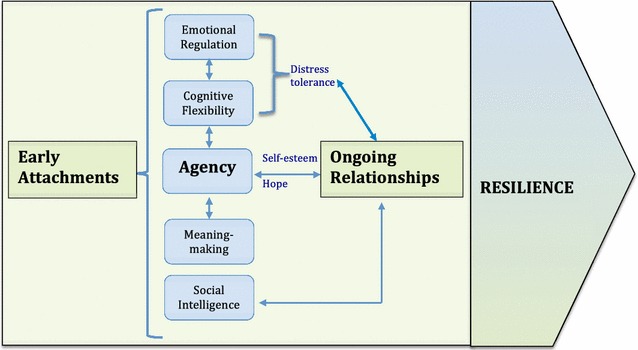
Proposed model for the interaction of factors contributing to the development and maintenance of resilience in post-conflict youth

Five essential factors interact to support the development of resilience in this population: emotion regulation, cognitive flexibility, agency, social intelligence and meaning-making (see Fig. 1). Cognitive flexibility and emotion regulation facilitate distress tolerance [8, 17] and development of healthy coping strategies [18–21]. Together they allow the preservation of one’s sense of agency in the face of stress or trauma. Both appear to have genetic and biological underpinnings [22–24]. They are further developed in the context of early attachments and continually mediated in relationships [25, 26]. In this population, children who could not self-regulate and self-soothe turned to substance use. During the war, children who were recruited into fighting forces were drugged. After the war, many continued using, and others started using as a way to escape from their traumatic memories [27]. These children described being addicted to drugs and were unable to envision a future for themselves. In contrast, children who were able to think creatively in the face of challenges and contain strong emotions could maintain a sense of control over their lives. From this, they derived self-esteem and a sense of hope for the future, which further enhanced their sense of control [8]. Moreover, because they were not using drugs or engaging in criminal activity, it was easier for them to build relationships with caring adults in the community because they embodied the values of these adults.

Cognitive flexibility, emotion regulation, agency, and social intelligence all contribute to building the capacity for relating. Children demonstrated this capacity by forming relationships, expressing empathy and behaving altruistically. Empathy strengthens one’s sense of connection. Altruism enhances self-worth while contributing to a sense of purpose or meaning [4]. A belief system can promote a sense of agency or it can reinforce a sense of helplessness. Contributing to one’s ability to form and maintain a positive belief system is cognitive flexibility [8, 17, 20]. Participants who demonstrated cognitive flexibility all had involved caregivers who were educated, suggesting that their cognitive capacities developed within these relationships. Relationships, in turn, further enhance agency by impacting self-esteem and hope, which further promote relationship-seeking [8].

There was a group of boys who were attending school who demonstrated greater adaptive functioning than their peers. While the girls in public school did not share this, some of the girls in private school did. At the private school, there were more girls than boys who participated in the study. A group of native Liberians who transcribed the interviews were then interviewed about their reactions to the data. They were not surprised by this finding. They explained that when families could only afford to send one child to private school, they would choose a girl because they believed she would be more protected there. There was an idea that girls needed to be protected from the risk of becoming pregnant and more generally from being corrupted or taken advantage of. Many of the more confident and ambitious boys in public school had previously attended private school and had switched to public school when their families faced financial hardship. Thus the girls in private school likely came from families that were similar to those of some of the boys attending public school.

In this population, adaptive functioning was only possible in the setting of supportive relationships and agency, both of which promoted other factors impacting resilience. While the literature does address the role of factors outside the individual in fostering resilience, the focus is on individual factors. This study highlights the importance of the individual’s capacity to build and sustain relationships with those around them and to access support in their communities. Relationships impact the development of all the individual factors that support resilience, including emotion regulation, cognitive flexibility, agency, social intelligence and meaning-making. Agency promotes an active coping style that leads children to take action to get their needs met, rather than “*following friends*,” a phrase many participants used to explain how they found themselves living on the streets and engaging in drug use. Rather than passively attaching themselves to other troubled children, those with an internal locus of control actively sought relationships that helped them to grow.

There were a number of limitations to this study. Participants were recruited from Monrovia only. Children in other parts of Liberia were not included, and the data may not be representative of their experiences. Interviews were conducted at a single time point, so resilience could not be assessed over time. No collateral information was obtained from teachers or family members. This allowed the children’s voices to come through, but it also meant that we were not able to obtain a complete picture of their circumstances. Some may have chosen, for a variety of reasons, to minimize the difficulties they were facing, while others may have chosen to magnify them. There is also the possibility of selection bias, as children who chose to participate may be different from those who did not.

## Conclusions

All children in Liberia were affected by the war in some way. Some fled the country, others witnessed the impact of the war on their family members, and many were exposed to violence themselves. Children who lost one or both parents during the war and were living on their own were less able to function post war. Many were not attending school and were engaging in substance use. Once they entered the world of substance use and other illegal activities, they became trapped. They were physiologically dependent on substances, and they had severed ties with their families and communities. These children in particular should be the focus of further study and intervention to target their substance use, while helping them to build sober communities for themselves.

Next steps toward helping these children include collaborating with organizations working with street children in order to help them reintegrate into the school setting. Identifying community leaders will be important for connecting these children with their communities. This would address the needs of children orphaned or separated from their families by the war and those orphaned by the Ebola epidemic. While children in school were generally functioning better, many were also struggling. A liaison between schools and families could identify children experiencing difficulties and offer additional support to those families. More mental health professionals are also needed, which will require the development of training programs in psychiatry, psychology and social work.
